# Road Surface Damage Detection Using Fully Convolutional Neural Networks and Semi-Supervised Learning

**DOI:** 10.3390/s19245501

**Published:** 2019-12-12

**Authors:** Chanjun Chun, Seung-Ki Ryu

**Affiliations:** Future Infrastructure Research Center, Korea Institute of Civil Engineering and Building Technology (KICT), Goyang 10223, Korea; chanjunchun@kict.re.kr

**Keywords:** road surface damage, semantic segmentation, autoencoder, convolutional neural network, semi-supervised learning

## Abstract

The various defects that occur on asphalt pavement are a direct cause car accidents, and countermeasures are required because they cause significantly dangerous situations. In this paper, we propose fully convolutional neural networks (CNN)-based road surface damage detection with semi-supervised learning. First, the training DB is collected through the camera installed in the vehicle while driving on the road. Moreover, the CNN model is trained in the form of a semantic segmentation using the deep convolutional autoencoder. Here, we augmented the training dataset depending on brightness, and finally generated a total of 40,536 training images. Furthermore, the CNN model is updated by using the pseudo-labeled images from the semi-supervised learning methods for improving the performance of road surface damage detection technique. To demonstrate the effectiveness of the proposed method, 450 evaluation datasets were created to verify the performance of the proposed road surface damage detection, and four experts evaluated each image. As a result, it is confirmed that the proposed method can properly segment the road surface damages.

## 1. Introduction

The various defects that occur on asphalt pavement are a direct cause of car accidents and countermeasures are required because they cause significantly dangerous situations. Among various defects, a pothole is a circular cavity formed by the internal damage of the asphalt concrete layer that fatigues and breaks the asphalt pavement surface. Such potholes usually occur during the rainy season of summer and early spring when the ground becomes weak due to freezing and thawing [1].

Potholes are dangerous for traffic safety because the water in the pothole makes it hard for the driver to perceive the fractured road surface. In particular, it is more difficult for drivers to recognize and avoid potholes on rainy nights when it is hard to secure a clear view. Fundamental countermeasures against pothole damage include improved asphalt pavement material and construction quality as well as the application of pavement sections that are suitable for the load of heavy vehicles. However, it is crucial to repair the roads as soon as possible after potholes are generated to prevent accidents in advance. In reality, however, it is difficult to detect and repair potholes in real time with limited human resources.

Various techniques have been proposed to detect potholes [1]. There are three major approaches: vibration sensor-based, laser scanning-based, and the computer vision-based methods. The vibration sensor-based method mainly uses sensors, such as acceleration sensors, that can detect the vibration amplitude. When using this method, the recognition performance may somewhat vary because the amplitude of the vibration may vary from vehicle to vehicle. It may also vary as damage on the road surface is difficult to detect without direct contact with the actual vehicle [2]. The laser scanning-based method can detect road surface damages with higher accuracy than other methods, but the size of the equipment is rather huge and relatively expensive. In addition, because such method scans roads at low speeds, it may also cause traffic congestion [3]. In terms of the computer vision-based method, the optical flow of the road surface image is used to detect damage to the road surface [4]. The optical flow is mounted on an embedded black box device to optimize and perform the computer vision-based algorithms in real time. The performance of the computer vision-based method may vary depending on the weather and time period due to the lower quality information comprising blurry images.

Image processing technologies have recently achieved significant progress with the emergence of deep neural networks. In particular, structures based on the convolutional neural network (CNN) is widely used among the various neural network structures [5]. CNN-based algorithms are rising in ImageNet Large Scale Visual Recognition Competition (ILSVRC), which addresses classification and detection problems [6]. The algorithms also achieved higher performance than conventional image processing algorithms in addressing regression problems [7], object detection [8], and semantic segmentation [9,10]. Among these, semantic segmentation is a neural network structure that divides the input image information into pixels or instances with their own meaning, and is only composed in the form of an autoencoder using only convolutional neural networks [10]. Therefore, it is also referred to as a fully convolutional neural network.

To detect the road surface damages, CNN-based techniques have also being studied [11,12]. They detect the road surface damages by using the object detection. The object detection is to find the position of an object on the image, which is in the form of bounding boxes, and to determine what class the object is [8]. In object detection, the parts of the road surface damages are not precisely segmented. In this paper, we focus on finding the road surface damages in the form of semantic segmentation.

Although convolutional neural networks show high performance on image processing in various applications, most approaches are limited to supervised learning [5]. Traditional machine learning methods can be divided into supervised and unsupervised learning categories [13], where supervised learning refers to the use of datasets that pair input data with labeled data to train models, and CNNs often use image information as input data. Labeled data can vary in segmentation, classification, and regression depending on the structure of the neural network, and much time and effort are required to acquire such labeled data. On the other hand, collecting unlabeled input data is a relatively easy and simple alternative to acquiring labeled data. Unsupervised learning refers to a type of machine learning algorithm used to generate new input data, or determine hidden structures from datasets consisting of input data without labeled responses [5]. In the case of performing supervised learning for the road surface damage detection technique based on the semantic segmentation, the labeled image that only segments road surface damages can be used as training data. In this paper, 5000 pieces of image datasets were collected, and these datasets must be labeled one by one to train the model, which requires a great deal of time and effort compared to collecting simple unlabeled input data.

This paper proposes a road surface damage detection technique using fully convolutional neural networks with semi-supervised learning. For this purpose, we collected training datasets through cameras installed in a vehicle while driving on an actual road in South Korea to train the CNN model. We segmented 6756 images, and the training dataset was increased to 40,536 images to enhance the performance by adjusting the brightness. Furthermore, to mitigate the difficulty of collecting these datasets for supervised learning and to improve the performance of the road surface damage detection technique, semi-supervised learning using pseudo labeling is utilized [14]. Semi-supervised learning is a method that uses the appropriate mix of labeled and unlabeled datasets. First, we train neural network models to detect road surface damage using labeled data. Several models are trained to form an ensemble, and road surface damage detection is performed on unlabeled image data. The neural network model regards the resulting images as pseudo-labeled data, and both the labeled data and pseudo-labeled data are used to train new CNN model again.

To demonstrate the effectiveness of the proposed method, 450 evaluation DB sets were created to verify the performance of the proposed road surface damage detection, and four experts evaluated each image. In addition, this paper compares the performance between a neural network model trained by using only the labeled datasets and a neural network model trained by using the labeled datasets as well as the pseudo-labeled datasets.

This paper is organized as follows. Section 2 describes the basic concept of a fully convolutional neural network. Section 3 describes the method of creating training datasets, the neural network structure, and the training method for the road surface detection technique using fully convolutional neural networks. Section 4 describes semi-supervised learning using pseudo labels for improving the performance. Section 5 validates the performance of the proposed road surface detection technique in which four experts evaluated each image. Section 6 presents the conclusion.

## 2. Fully Convolutional Neural Networks

**Figure 1:**
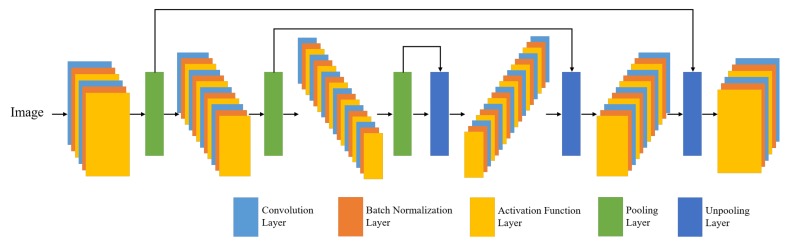
Structure of fully convolutional neural network.

This Section describes the structure of a fully convolutional neural network. As shown in Figure 1, fully convolutional neural networks are composed of convolutional neural networks without fully connected networks (FCN). They are also referred to as a deep convolutional autoencoders. In the characteristics of the autoencoder, when a certain input is given to the neural network, it becomes smaller and then returns to its original input size. Therefore, it is called an autoencoder because it is similar to the encoding and decoding procedures [15,16]. This neural network structure is widely used in semantic segmentation to classify images into pixel-by-pixel classification [9,10]. The following subsections describe the various layers that are used in fully convolutional neural networks in detail.

### 2.1. Convolutional Neural Network (CNN)

**Figure 2:**
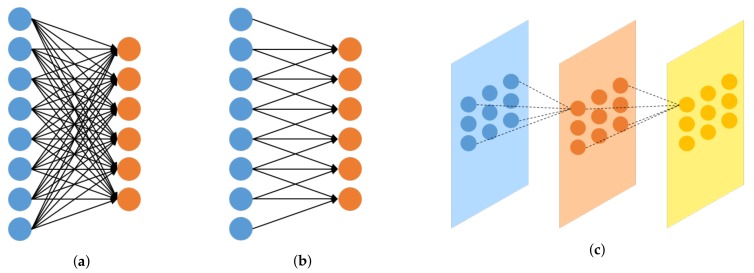
Examples of: (**a**) fully connected neural network (FCN) and (**b**) 1D and (**c**) 2D convolutional neural networks (CNNs): all neurons are connected in (**a**), while only adjacent neurons are connected in (**b**,**c**).

The convolutional layer is shown in Figure 2. Figure 2a shows the fully connected neural network, and Figure 2b,c illustrate 1D and 2D convolutional neural networks, respectively. In a fully connected neural network, all of the neural networks in the previous layer and the next layer are connected to each other. On the other hand, in the convolutional neural network, only the adjacent units are connected. Fully connected neural networks must be converted into one dimension, which leads to the loss of spatial information. As convolutional neural networks can prevent this loss, they are widely used when using 2D or 3D image data [17]. Computation proceeds by shifting a filter of a certain size at regular intervals, which is referred to as a stride. The size of the next layer is influenced by the stride. Specifically, the size of the next layer is determined by the pooling layer, stride, and zero padding [17]. In this paper, we only apply zero padding and stride without any pooling layer.

### 2.2. Deconvolutional Neural Network

A neural network that reverses the convolutional neural network is referred to as a deconvolutional neural network [18]. The size of the output layer from the convolutional neural network and the size of the input layer of the deconvolutional neural network are the same when the same pooling, stride, and zero padding sizes are applied. Furthermore, the size of the input layer of the convolutional neural network and the size of the output layer of the deconvolutional neural network are also the same. As the convolutional neural network can be paired with the pooling layer, the deconvolutional neural network is often paired with the un-pooling layer. The pooling layer can achieve the effect of downsampling, while the un-pooling layer can have an upsampling effect. However, this paper does not place a pooling layer in the convolutional layer and also does not insert an un-pooling layer. Instead, the convolutional and deconvolutional neural networks are configured with zero padding and stride.

### 2.3. Batch Normalization and Activation Function

Batch normalization is one of the techniques to accelerate the convergence rate of learning, and is usually placed between the affine layer and the activation function [19]. Batch normalization reduces the level of dependence on the initial value and prevents overfitting without performing dropout or regularization [20]. This paper performs batch normalization before the activation function after the convolutional layer and deconvolutional layer. As for the activation function, we use the rectified linear unit (ReLU), which is commonly used, and is referred to as f(x)=max(0,x) [20].

### 2.4. Skip Connections

In the form of autoencoder neural networks, skip connections are widely utilized [21]. In Figure 1, the skip connections are directly connected from the encoder stage to the decoder stage. The loss of specific image information gradually increases through the convolutional and the pooling layers. When the network gets deeper from the convolutional or pooling layers, this situation can be even more highlighted. To reduce this problem, skip connections are utilized to concatenate the CNN feature in the encoder stage to the CNN feature in the decoder stage. Skip connections can also reduce the vanishing gradients that can occur when designing deep networks.

## 3. Road Surface Damage Detection

This section describes the road surface damage detection technique using fully convolutional neural networks. The first subsection describes the training datasets created and labeled to train the neural network. The second subsection describes the structure and training method of the fully convolutional neural networks.

### 3.1. Creating the Training DB

We collected training datasets through cameras installed on a vehicle while driving on an actual road in South Korea to train the neural network model to automatically detect road surface damages. The photographs were taken while driving up to a maximum speed of 100 km/h on expressways and city roads. The photographs were taken at a resolution of [1920 × 1080], by installing a smartphone in a location where the black box is usually installed.

**Figure 3:**
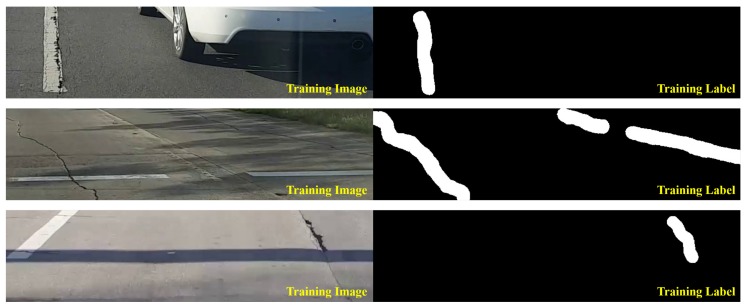
Examples of training sets for road surface damage detection technique.

**Figure 4:**
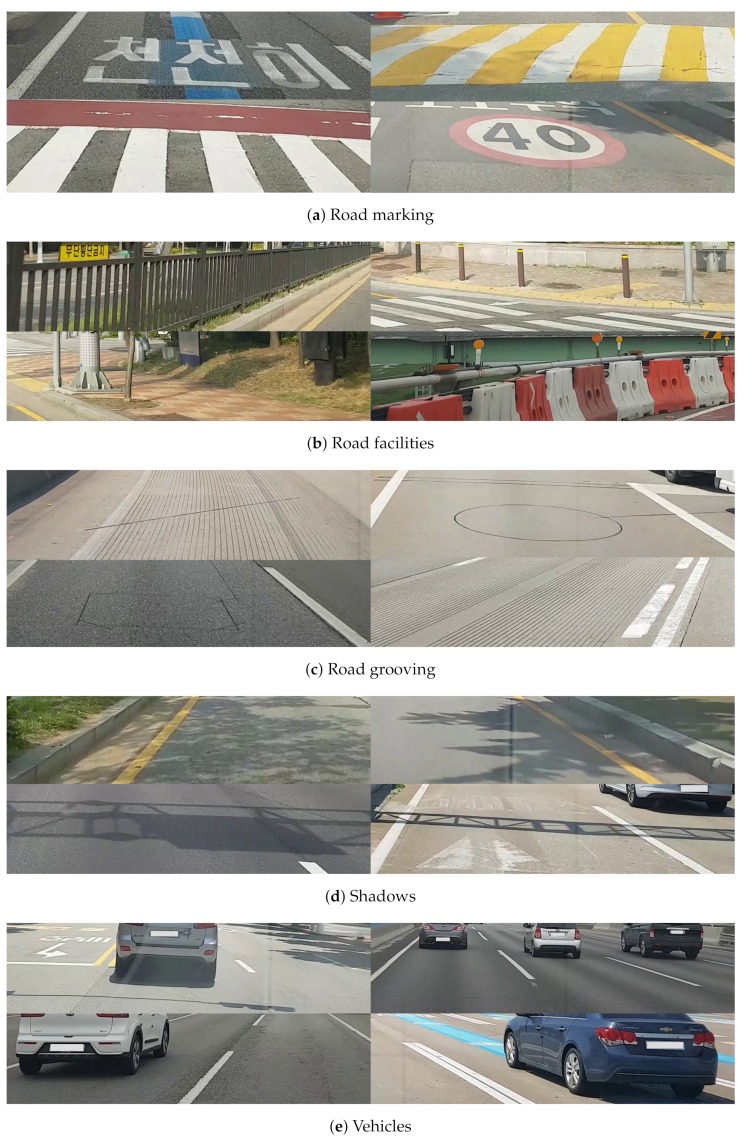
Examples of training images depending on the class. At least one image includes one of six classes (including road surface damage class), and is classified into a class that is considered more important if it includes several classes.

Figure 3 shows labeled image examples to detect road surface damage. As shown in the figure, the part of the input image with road surface damage appears as white, and the remaining parts in the image appear as black. Note that the damaged part of the road surface is represented by the white pixel value of 255, and the other parts are represented by the black pixel value of 0 solely for visualization. In the actual training process, they were mapped as 1 and 0, respectively. Only [800 × 200] size was cropped from the FHD image. When the camera is installed in the form of a black box inside a vehicle, the captured image generally includes the bonnet of the vehicle. In addition, road surface damage at a long distance from the vehicle is difficult to judge from the captured image. The same issue exists in the next lane. Therefore, in this paper, [800 × 200] size was only cropped from the [1920 × 1080] size. When cropping to 800 pixels wide, one lane was typically included. In this paper, a total of 6756 images were collected to train the neural network model. From these images, about 47% includes road surface damages. Other images include images of vehicles, shadows, road markings, road facilities, and road grooving, which could be mistaken for road surface damages. Table 1 shows the number of images corresponding to each class. The numbers of road marking, road facilities, road grooving, shadows, vehicles, and road surface damages are 1260, 587, 599, 451, 681 and 3178, respectively. At least one image includes one of six classes, and is classified into a class that is considered more important if it includes several classes. If an image contains both road surface damages and another class, the image is classified as a road surface damage class. Figure 4 shows the examples of training images depending on the class except for the road surface damage class. In the case of road marking class, they include pedestrian crossings, signs, and symbols that appear on the road surface. Road facilities refer to railings, street lamps, and beacons. Road grooving includes grooving, which prevents slipping, and signs to mark the burial. Shadows and vehicles refer to shadows caused by various structures or trees and vehicles in the ROI area.

**Figure 5:**
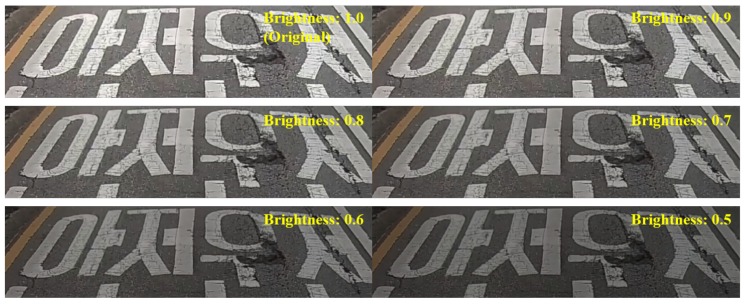
Examples of training images depending on brightness.

The training images are all captured at bright sunny days. Therefore, the training images were augmented by adjusting the brightness to generate robust performance even in dark environments, as shown in Figure 5. Six levels were adjusted, including the original, and then the number of images increased from 6756 to 40,536.

### 3.2. Neural Network Architecture

**Figure 6:**
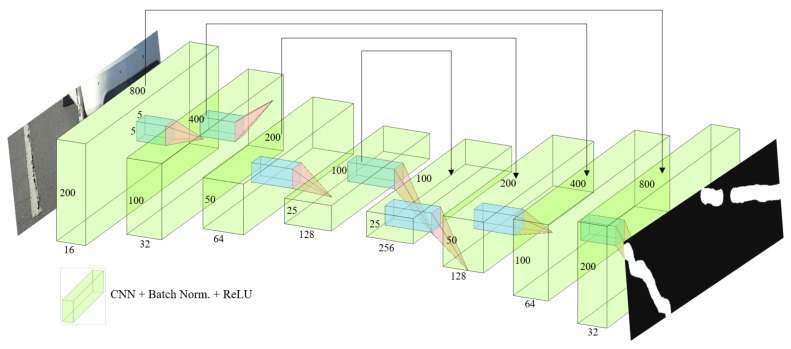
Overall architecture of fully convolutional neural networks for the road surface damage detection technique.

Figure 6 shows the neural network architecture for road surface damage detection technique. The overall structure has eight hidden convolution layers excluding the input and the output layers. Four of these layers are stridden convolution layers, and the other four are stridden deconvolution layers. We used a stride [2 × 2] for the convolution and deconvolution layers without any pooling and un-pooling layer. Furthermore, the kernel size is [5 × 5]. The numbers of convolution filters are 16, 32, 64 and 128, respectively. The numbers of deconvolution filters are 256, 128, 64 and 32, respectively. The numbers of filters in the deconvolution layers are twice as large as the numbers of filters in the convolution layers because the skip connections are utilized to reduce the loss of specific image information. Except for the absence of a pooling and un-pooling layer, there is no significant difference from the neural network architecture shown in Figure 1. The loss function was configured in the form of mean square error (MSE) [22], such that

(1) $$J\left(\theta \right)=\frac{1}{M}\sum_{i=1}^{M}{\left(y_{i}-h_{\theta }\left(x\right)\right)}^{2}$$

where $y_{i}$ indicates the actual labeled value, and $h_{\theta }$ means the neural network output for input *x*. Adaptive moment estimation (Adam) was used as the optimization technique [23]. Adam stores both exponentially decaying average of past gradients and squared gradients, such that

(2) $$\theta_{t+1}=\theta_{t}-\frac{\eta }{\sqrt{v_{t}+\epsilon }}\frac{\sqrt{1-\beta_{2}^{t}}}{1-\beta_{1}^{t}}m_{t}$$

where

(3) $$\begin{array}{c} m_{t}=\beta_{1}m_{t-1}+\left(1-\beta_{1}\right)g_{t}, \end{array}$$

(4) $$\begin{array}{c} v_{t}=\beta_{2}v_{t-1}+\left(1-\beta_{2}\right)g_{t}^{2}. \end{array}$$

**Figure 7:**
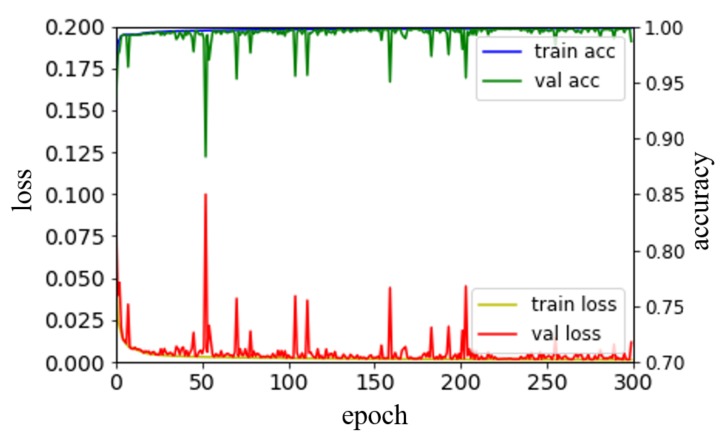
Loss value and accuracy according to epoch for training and validation sets.

Here, $\beta_{1}$ and $\beta_{2}$ were set to 0.9 and 0.999, respectively. As described in Section 2, the ReLU function was used as the activation function [20], and batch normalization was performed between the convolution layer and the activation function [19]. Figure 7 shows the loss value according to the training iteration. From the 6756 images, 1351 images (about 20%) were used as the validation datasets, where the validation images were randomly selected. The number of epochs was limited to 1000, and the model with the lowest validation loss value was used during training.

**Figure 8:**
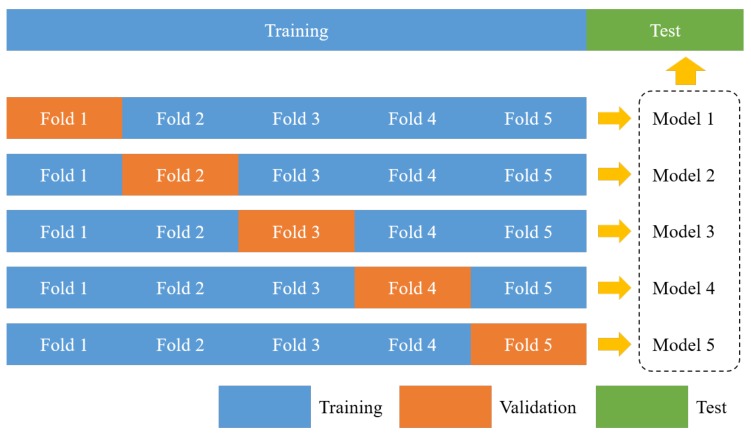
Ensemble using K-fold cross validation (*K* = 5).

**Figure 9:**
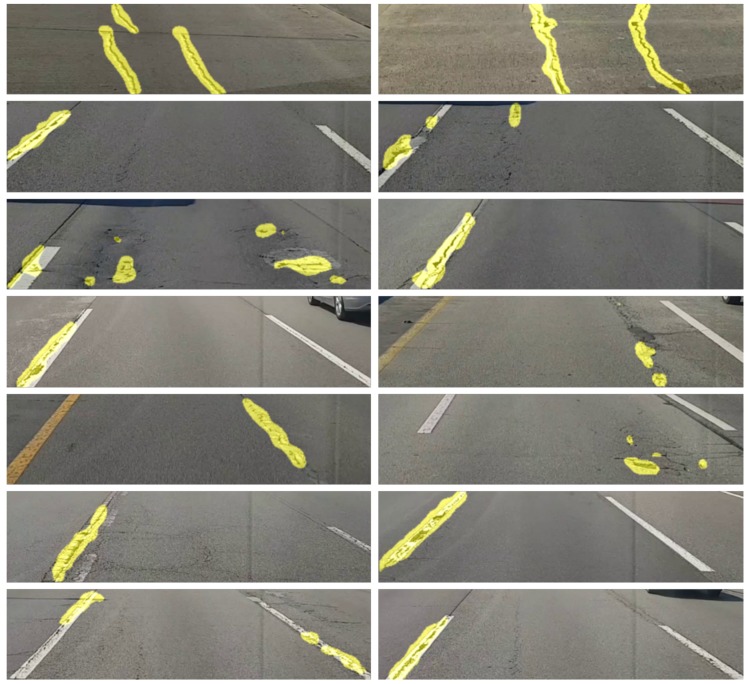
Results of the proposed road surface damage detection.

To improve the performance of the trained model, five models were generated and an ensemble was formed [24]. Ensemble refers to using not only one model that shows the best performance among several trained models, but also a combination of results obtained from several different models. It is also possible to harmonize various machine learning methods other than the deep neural network. In this paper, the ensemble was formed using only the models obtained by repeatedly training the proposed neural network architecture. In particular, an ensemble was formed through the *K*-fold cross validation technique, where *K* was set to 5. *K*-fold cross validation technique, shown in Figure 8, divides the training dataset into *K* equivalent datasets. In addition, K−1 datasets are used for training, and the other one is used for validation. A total of *K* models can be generated according to which dataset is used for validation among the *K* datasets. In this paper, we set *K* to 5 so that the validation dataset was distributed to 20%. Therefore, five models were generated. Figure 9 shows the results of the proposed road surface damage detection. As shown in the figure, it is confirmed that only the damaged parts of the road surface are segmented.

## 4. Semi-Supervised Learning Using Pseudo Labels

Semi-supervised learning refers to the use of a large number of unlabeled datasets and a relatively smaller number of labeled datasets [14]. A large number of unlabeled datasets is pseudo-labeled to obtain a large dataset. It would be perfect to have a very large number of labeled datasets, but it takes a lot of time and effort to build them. Accordingly, in a practical problem, there are many cases where a relatively small amount of dataset only exists. We wanted to segment all of the classes, shown in Table 1, but we only segmented the road surface damages because it takes a lot of time and effort.

**Figure 10:**
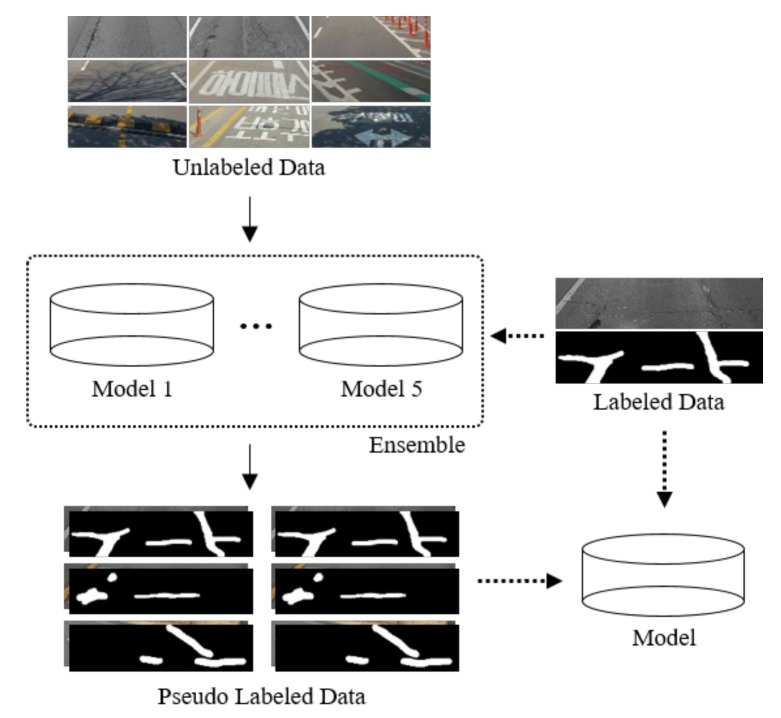
Overall structure of road surface damage detection using pseudo labels.

Figure 10 presents the overall structure of the semi-supervised learning method using the pseudo labels to detect road surface damage. First, the model was trained to perform road surface damage detection, as described in Section 3, using labeled data. In addition, a five models were generated to improve the performance of the model. Using these models, 61,811 unlabeled image datasets were entered as input from the ensemble of the trained models to obtain the predicted output image datasets. Such predicted output image datasets were regarded as pseudo-labeled image datasets. Therefore, 6756 labeled datasets and 61,811 unlabeled datasets were utilized. In total, 68,567 dataset were used to retrain the neural network model. Compared to our computing power, thus is a large number of datasets. Thus, the brightness was reduced to three levels instead of six levels. An ensemble would be able to generate more sophisticated results, but this method requires a lot of training time. Therefore, we did not apply an ensemble in the last step.

**Figure 11:**
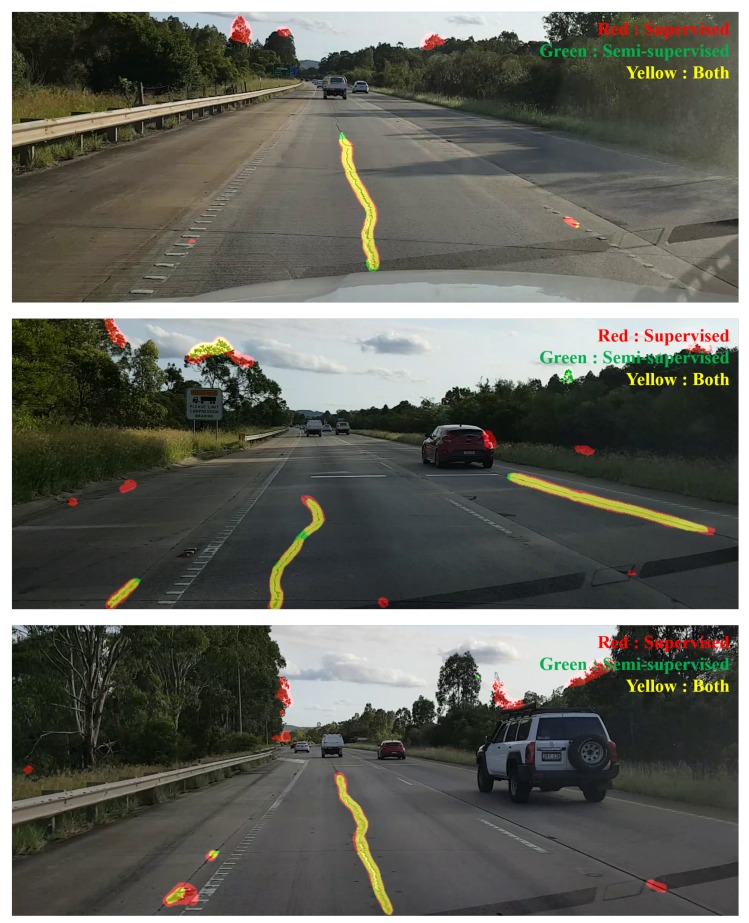
Results of road surface damage detection using pseudo labels.

Figure 11 shows the results of models using supervised learning and semi-supervised learning. Note that all models have [800 × 200] image data, but the ROI is larger. This is because the neural network model consists only of CNNs, and there is no fully connected network. In the figure, the area in red is estimated from supervised learning, without using the existing pseudo-labeled datasets. The area in green is from semi-supervised learning using the labeled datasets as well as the pseudo-labeled datasets. The area in yellow is estimated from both models. Consequently, the combination of red and yellow is the result of supervised learning, and the combination of green and yellow is the result of semi-supervised learning. As shown in the figure, it can be confirmed that there are many false positives in supervised learning. Semi-supervised learning also has false positives, but relatively fewer than supervised learning.

## 5. Performance Results and Evaluation

**Figure 12:**
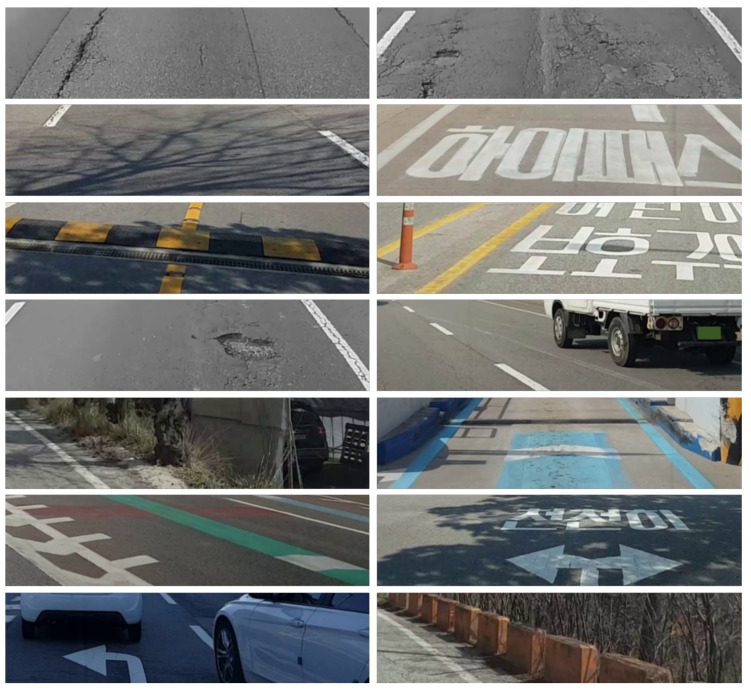
Examples of evaluation images for road surface damage detection technique.

To demonstrate the effectiveness of the proposed road surface damage detection, a subjective performance evaluation was performed. In semantic segmentation problems, it is common to measure the mean intersection over union (mIOU) to compare performance [25]. In this evaluation, the labeled datasets used for training were not finely segmented, and considerably coarse. Since the predicted results are also very coarse, the subjective evaluation was performed rather than measuring the mIOU. Note that there was no overlap with training, and it consisted only of an image dataset that was considered more difficult than training. Figure 12 shows example images of the dataset used for the evaluation. Such images include not only the road surface damages but also road marking, road facilities, road grooving, shadows, and vehicles.

Four experts subjectively evaluated the 450 images, which were displayed randomly. All four evaluators who analyzed the images are experts that work in the field of road traffic. The experts evaluated each image and matched them with the closest item, as shown in Table 2. When there was road surface damage in the image, the cases were divided by whether or not the proposed model properly segmented the parts with road surface damage. When there was no road surface damage in the image, the cases were divided by whether or not the parts with road surface damage were not properly segmented. This is an evaluation method widely used for statistically evaluating the performance of algorithms and is used in many areas [26].

Table 2 also shows the result of evaluating the proposed road surface damage detection technique. First, the evaluation criteria were divided into Tp, Tn, Fp, and Fn, which indicate the numbers of true positives, true negatives, false positives, and false negatives, respectively. In this paper, Tp corresponds to the case when the area that needs to be segmented as road surface damage is accurately segmented. Each expert subjectively reviewed the resulting images from the trained model segmenting road surface damages, and evaluated them as Tp, Tn, Fp, and Fn. From the four indicators, we can derive the precision, recall, accuracy, and F_1_-score [26]. First, the accuracy increased from 0.8728 to 0.9387. In addition, the precision significantly increased from 0.7012 to 0.9014. It is implied that the performance of detecting false positives has improved significantly. As shown by the number of false positives, there were 193 Fp in supervised learning, but the number dramatically decreased to 50 in semi-supervised learning. On the other hand, the recall decreased from 0.9264 to 0.8822. Fortunately, only a relatively small number decreased compared to the increase in precision. The recall indicates how well the number of actual positives is classified as positive, which is closely related to Fn. There were 36 Fn in supervised learning, and the number increased to 61 in semi-supervised learning. Finally, the F_1_-score improved from 0.7982 to 0.8917. Although the evaluation of the recall decreased slightly, the overall F_1_-score was higher than that of supervised learning because the precision criterion improved significantly. In the future, a more robust form of neural network model is required, which can be obtained by securing various training methods and high-quality datasets.

## 6. Conclusions

We propose the road surface damage detection technique using fully convolutional neural networks. The proposed neural network architecture is in the form of a deep convolutional autoencoder that is mainly used for semantic segmentation. In total, 6756 labeled datasets were collected by segmenting and classifying into six classes using the images obtained by driving on actual roads. The dataset was augmented by adjusting the brightness in six levels, and the performance of the model was stabilized by forming an ensemble using *K*-fold cross validation. Furthermore, to mitigate the difficulties of collecting input and labeled datasets for supervised learning, this paper proposes a road surface damage detection technique based on semi-supervised learning using pseudo-labeled datasets. The neural network model regarded the predicted images as pseudo-labeled data, and both the labeled data and pseudo-labeled data were used to train the neural network model again. In terms of subjectively evaluating the performance of the trained model, the precision criterion significantly improved compared to that of supervised learning, while the recall criterion slightly decreased. However, the overall F_1_-score was also higher than that of supervised learning. In the future, a more robust form of neural network model is required by securing various training methods and high-quality datasets.

## Figures and Tables

**Table 1:** Numbers of training DB for road surface damage detection technique.

	Marking	Facilities	Grooving	Shadows	Vehicles	Damages	Total
# of images	1260	587	599	451	681	3178	6756
Percentage	18.7%	8.7%	8.9%	6.7%	10.1%	47.0%	100.0%

**Table 2:** Subjective evaluation result by four experts.

		Tp	Tn	Fp	Fn	Precision	Recall	Accuracy	F_1_-Score
Supervised	Expert I	112	281	47	10	0.7044	0.9180	0.8733	0.7972
	Expert II	105	263	77	5	0.5769	0.9545	0.8178	0.7192
	Expert III	125	263	51	11	0.7102	0.9191	0.8622	0.8013
	Expert IV	111	311	18	10	0.8605	0.9174	0.9378	0.8880
	**Total**	**453**	**1118**	**193**	**36**	**0.7012**	**0.9264**	**0.8728**	**0.7982**
Semi- supervised	Expert I	119	308	13	10	0.9015	0.9225	0.9489	0.9119
	Expert II	111	309	14	16	0.8880	0.8740	0.9333	0.8810
	Expert III	119	313	13	15	0.9015	0.8881	0.9391	0.8947
	Expert IV	108	312	10	20	0.9153	0.8438	0.9333	0.8781
	**Total**	**457**	**1242**	**50**	**61**	**0.9014**	**0.8822**	**0.9387**	**0.8917**
